# Emerging Biomarkers in Glioblastoma

**DOI:** 10.3390/cancers5031103

**Published:** 2013-08-22

**Authors:** Mairéad G. McNamara, Solmaz Sahebjam, Warren P. Mason

**Affiliations:** Pencer Brain Tumor Centre, Princess Margaret Cancer Centre, 610 University Avenue, Toronto, Ontario M5G 2M9, Canada; E-Mails: mairead.mcnamara@uhn.on.ca (M.G.M.N.); ssahebjam@gmail.com (S.S.)

**Keywords:** glioblastoma, molecular classification of glioblastoma, molecular biomarkers, imaging biomarkers

## Abstract

Glioblastoma, the most common primary brain tumor, has few available therapies providing significant improvement in survival. Molecular signatures associated with tumor aggressiveness as well as with disease progression and their relation to differences in signaling pathways implicated in gliomagenesis have recently been described. A number of biomarkers which have potential in diagnosis, prognosis and prediction of response to therapy have been identified and along with imaging modalities could contribute to the clinical management of GBM. Molecular biomarkers including O(6)-methlyguanine-DNA-methyltransferase (MGMT) promoter and deoxyribonucleic acid (DNA) methylation, loss of heterozygosity (LOH) of chromosomes 1p and 19q, loss of heterozygosity 10q, isocitrate dehydrogenase (IDH) mutations, epidermal growth factor receptor (EGFR), epidermal growth factor, latrophilin, and 7 transmembrane domain-containing protein 1 on chromosome 1 (ELTD1), vascular endothelial growth factor (VEGF), tumor suppressor protein p53, phosphatase and tensin homolog (PTEN), p16INK4a gene, cytochrome c oxidase (CcO), phospholipid metabolites, telomerase messenger expression (hTERT messenger ribonucleic acid [mRNA]), microRNAs (miRNAs), cancer stem cell markers and imaging modalities as potential biomarkers are discussed. Inclusion of emerging biomarkers in prospective clinical trials is warranted in an effort for more effective personalized therapy in the future.

## 1. Introduction

Glioblastoma (GBM), the most common primary malignant brain tumor, is a morphologically heterogenous tumor type. The median survival of patients with GBM in clinical trial populations treated with multimodal treatment approaches is approximately 15 months, with only 3%–5% of patients surviving longer than 36 months [1]. However, survival varies amongst GBM patients [2], therefore distinct morphologic features within this, the most aggressive high grade glioma, may result in differing responses to standard combined chemo-radiotherapy with temozolomide [3].

New information on glioma susceptibility loci and genetic signatures (Table 1) are constantly becoming available. Single nucleotide polymorphisms associated with susceptibility to gliomas have been identified (rs2736100 [telomerase messenger expression (TERT)], rs11979158 and rs2252586 [epidermal growth factor receptor (EGFR)], rs4295627 [CCDC26], rs498872 [PHLDB1], rs6010620 [RTEL1], and rs4977756 [CDKN2A/CDKN2B]).

**Table 1:** Some described genetic signatures in glioblastoma.

Verhaak classification [4]	Phillips classification [5]	Jiao classification [6]
Classical—High *EGFR*, *TP53*, longest survival of subgroups in response to aggressive treatment.	Proliferative—Enriched for neural stem cell markers, *PTEN* loss, *EGFR* amplified or normal, Akt (protein kinase B) cell signaling pathway activation, shorter survival than proneural subgroup.	I-X glioma—GBM like; multiple molecular subgroups, distinct from *IDH1/ATRX/TP53* (I-A glioma) and *IDH/CIC/FUBP1* (I-CF glioma) tumors—prognosis approximately 1 year.
Proneural—*TP53* mutated, *IDH1* gene mutated, *PDGFRA* mutated, patients significantly younger.	Proneural—*PTEN* intact, *EGFR* normal, Notch activation, longer survival than proliferative and mesenchymal subgroup.	
Mesenchymal—*NF1* mutated, *TP53* mutated, *PTEN* mutated.	Mesenchymal—Enriched for neural stem cell markers, *PTEN* loss, *EGFR* amplified or normal, Akt cell signaling pathway activation, shorter survival than proneural subgroup.	
Neural—mutations in many of same genes as the other 3 subgroups. Oldest patients on average.		

EGFR, epidermal growth factor receptor; TP53, tumor suppressor protein 53; IDH1, isocitrate dehydrogenase 1; PDGFRA, platelet derived growth factor receptor A; NF1, neurofibromatosis type 1; PTEN, phosphatase and tensin homolog; ATRX, alpha thalassemia/mental retardation syndrome X-linked mutation; CIC, homolog of *Drosophila capicua*; FUBP1, far-upstream binding protein 1.

TERT and RTEL1 risk alleles have been found to be associated with high-grade disease [7]. A tripartite genetic signature was described in 2012 [6] with I-X tumors being described as similar to GBM with poor patient survival, advanced age and lacking in isocitrate dehydrogenase (IDH) and Alpha Thalassemia/Mental Retardation Syndrome X-linked (ATRX) mutations and therefore being distinct from *IDH1/ATRX/TP53* (I-A) and IDH1/homolog of *Drosophila capicua* (*CIC*)/far-upstream binding protein 1 (FUBP1) (I-CF) tumors. Patients with I-X tumors are further classified into prognostic groups on the basis of other mutational, copy number, gene expression, and Deoxyribonucleic Acid (DNA) methylation alterations as will now be described.

Phillips *et al*. described three molecular subclasses of high grade glioma; proneural, proliferative and mesenchymal. The proneural subclass was phosphatase and tensin homolog (PTEN) intact, epidermal growth factor receptor (EGFR) normal, had notch activation and longer survival whereas the proliferative and mesenchymal group, both enriched for neural stem cell markers, had PTEN loss, were *EGFR* amplified or normal and had Akt (also known as protein kinase B) cell signaling pathway activation with shorter survival [5].

More recently, the Cancer Genome Atlas (TCGA) Network further defined the molecular classification of GBM and described four subtypes; classical, proneural, mesenchymal and neural [4]. Classical GBM tumors were characterized by high levels of EGFR and *TP53* was not found to be mutated in classical GBM (TP53 is the most frequently mutated gene in GBM and is normally responsible for suppressing tumor growth). The classical subgroup survived the longest of the subgroups in response to aggressive treatment.

*TP53* was found to be significantly mutated in proneural tumors as were mutations in the *IDH1* gene. Platelet derived growth factor receptor A (PDGFRA), which plays an important role in cell proliferation, cell migration, and angiogenesis, was found to be mutated and expressed in abnormally high amounts only in proneural tumors and not in any other subgroups. Patients in the proneural subgroup were found to be significantly younger, but those who received aggressive treatment did not survive significantly longer than proneural patients who did not receive aggressive treatment.

The mesenchymal subgroup contained the most frequent number of mutations in the neurofibromatosis type 1 (*NF1*) tumor suppressor gene and frequent mutations in the *PTEN* and *TP53* tumor suppressor genes also occurred. The mesenchymal group had an improvement in survival after aggressive treatment, unlike those in the proneural, and neural subgroups.

The neural subgroup had mutations in many of the same genes as the other groups and were the oldest patients, on average. The improvement in survival after aggressive treatment was not as much as in the classical and mesenchymal groups.

The knowledge of genomic changes that drive GBM gleaned from the TCGA in addition to other studies on potential molecular predictive and prognostic biomarkers in GBM including MGMT promoter and DNA methylation, LOH of chromosomes 1p and 19q, LOH 10q, IDH mutations, EGFR, ELTD1, VEGF, tumor suppressor protein p53, PTEN, p16INK4a gene, CcO, phospholipid metabolites, telomerase messenger expression (hTERT mRNA), miRNAs, cancer stem cell markers and imaging modalities as potential biomarkers are discussed and their role, if any, in clinical decision making will now be discussed (Table 2).

**Table 2:** Molecular and metabolic alterations in GBM and their potential biomarker status.

Molecular/metabolic alteration	Possible biomarker status
O(6)-methlyguanine-DNA-methyltransferase (MGMT) promoter methylation	Prognostic, predictive [1]
Loss of heterozygosity chromosome 1p 19q	No prognostic significance [8]
Loss of heterozygosity 10q	Prognostic [9]
Isocitrate dehydrogenase (IDH) mutational status	Prognostic [10]
Epidermal growth factor receptor (EGFR)	Prognostic [11]
Epidermal growth factor, latrophilin, and 7 transmembrane domain-containing protein 1 on chromosome 1 (ELTD1)	Diagnostic, potentially prognostic [12]
Vascular endothelial growth factor (VEGF)	Potentially prognostic [13]
Tumor suppressor protein p53	Diagnostic [14]
Phosphatase and tensin homolog (PTEN)	Prognostic, possibly predictive [13]
p16INK4a gene	Inconsistent findings [13]
Cytochrome c oxidase (CcO)	Potentially prognostic [15]
Phospholipid metabolites	Potentially predictive [16]
Telomerase messenger expression (hTERT messenger ribonucleic acid [mRNA])	Potentially diagnostic [17], prognostic [18]
microRNAs (miRNAs)	Diagnostic, prognostic [19]
Cancer stem cell markers	Potentially prognostic [20,21]

## 2. Molecular and Metabolic Alterations in GBM and Their Potential Biomarker Status

### 2.1. *MGMT* and DNA Methylation

The *MGMT* gene is located at chromosome 10q26 and encodes a DNA repair protein that removes the alkyl groups from the O6 position of guanine, which are commonly produced by chemotherapeutic alkylating agents. Epigenetic silencing of the *MGMT* DNA repair gene by promoter methylation compromises DNA repair. The methylation status of *MGMT* was determined retrospectively from the tumor tissue of 206 patients [22] who were enrolled in the randomized phase III trial where patients with a diagnosis of GBM were treated with concomitant and adjuvant temozolomide and radiotherapy [3]. Irrespective of treatment, *MGMT* promoter methylation was an independent favorable prognostic factor (Hazard Ratio (HR) 0.45 (95% confidence interval (CI) 0.32–0.61) *p* < 0.001). Median overall survival was reported to be best in patients with a methylated promoter treated with temozolomide and radiotherapy (23.4 months *vs.* 15.3 months in the radiotherapy alone group) [1] and thus was reportedly the first predictive biomarker in brain tumors and potentially allows selection of patients who benefit from treatment with temozolomide and radiotherapy but is not of assistance in diagnostics.

However, to date, *MGMT* promoter hypermethylation does not guide treatment strategies for patients with GBM but may provide valuable prognostic information. In addition, there is a lack of standardization in the measurement of MGMT with various available molecular testing platforms available and consequently there are highly variable results. Unfortunately, no single method of MGMT analyses has emerged as a clear choice for routine clinical testing of MGMT in glioma patients. Methylation analyses is favored, but the resulting expense and inaccessibility are barriers to use in general clinical practice [23]. There is the necessity for the development of high throughput, sensitive, inexpensive and reliable assays and more research into immunohistochemistry is warranted as an alternative to methylation analyses.

Promoter DNA methylation alterations were profiled in 272 GBM tumors in the context of the TCGA and a distinct subset of samples displaying concerted hypermethylation at a large number of loci were found. These glioma-CpG island methylator phenotype (G-CIMP) tumors belonged to the proneural subgroup and were tightly associated with *IDH1* somatic mutations, and patients were younger at the time of diagnosis and had improved outcomes [24]. This finding was confirmed in another study where genome-wide DNA methylation profiling of short-term survivors (<1 year) and long-term survivors (>3 years) again demonstrated that a subset of long-term survivors showed a G-CIMP positive phenotype that was tightly associated with *IDH1* mutation status and was confirmed by analysis of the G-CIMP signature genes [25].

### 2.2. Loss of Heterozygosity (LOH) of Chromosomes 1p and 19q

In GBM, deletions involving 1p and 19q are uncommon but have been identified in <10% of diffuse astrocytic gliomas (including GBMs) [26]. In contrast, LOH of chromosomes 1p and 19q is the most common genetic alteration in oligodendroglioma tumors and is associated with favorable response to chemotherapy, radiation and survival [27]. A study involving central pathology review of the European Organisation for Research and Treatment of Cancer (EORTC)_26981/National Cancer Institute of Canada (NCIC)_CE.3 trial which specifically addressed whether 360 patients with GBM displaying distinct morphologic features respond differently to combined chemo-radiotherapy with temozolomide, with particular focus on the presence of an oligodendroglioma-like component, demonstrated that GBM with an oligodendroglioma-like component (GBM-O) represented 15% of all confirmed GBM and was not associated with a more favorable outcome. Co-deletion of 1p19q was found in only one case while GBM-O significantly enriched for *IDH1* mutations and *EGFR* amplifications compared with other GBM. Expression profiles classified most of the GBM-O into two subtypes, 36% as proneural and 43% as classical GBM and concluded that recognition of an oligodendroglioma-like component in an otherwise classic GBM identifies a pathogenetically mixed group without prognostic significance [8]. However, this study did report that the detection of pseudo-palisading necrosis (PPN) was associated with benefit from chemotherapy, while no such effect was present in the absence of PPN [8].

### 2.3. Loss of Heterozygosity 10q

Allelic deletions encompassing all or part of chromosome 10q have been reported as a frequent genetic alteration in the pathways to primary and secondary GBM [28], indicating that loss of one or more tumor suppressor genes on 10q plays a role in GBM formation. One of these genes is PTEN, a gene on 10q23 which encodes a dual-specificity protein phosphatase [29]. Lower losses on chromosome 10 are seen in anaplastic astrocytoma indicating that loss of heterozygosity on chromosome 10 is a terminal genetic event associated with GBM [30]. Loss of heterozygosity on chromosome 10q has been found to be associated with reduced survival of GBM patients [9] and in a study of 25 cases of GBM in Indian patients was seen more frequently in older patients [31]. The loss of heterozygosity profile may thus have prognostic implications but to date has not guided treatment options.

### 2.4. IDH

IDH catalyzes the conversion of isocitrate into α-ketoglutarate within the citric acid cycle. IDH1 and IDH2 are involved in a number of metabolic processes such as signal transduction, lipid synthesis, oxidative stress, and oxidative respiration [26].

For all gliomas, patients harboring *IDH* mutations appear to have a prognostic advantage compared with patients without *IDH* mutations [10,26]. Specifically, a study by Jansen *et al.*, [26] reported that somatic mutations were present in 18 of 149 (12%) GBMs and seemed to correlate with increased survival. The overall survival in patients with IDH mutations was 31 months in comparison to 15 months in those without *IDH* mutations [10]. Despite IDH being a useful prognostic tool, it currently does not appear to be able to predict responsiveness to a particular type of therapy.

### 2.5. EGFR

EGFR is a common molecular hallmark of GBM and promotes a pro-proliferative signal [32]. *EGFR* amplification at 7p12 is the most commonly amplified and over expressed gene in primary GBM (30%–70%) with EGFRvIII being the most prominent mutated receptor tyrosine kinase receptor in GBM, occurring in approximately 50% of GBM cases that overexpress EGFR [26].

EGFRvIII constitutively activates the EGFR-PI3K pathway [10]. It has been reported that GBMs harboring constitutively active EGFRvIII receptors display a more invasive phenotype than those with wild-type EGFR [32] but the efficacy of EGFR inhibitors remains controversial in newly diagnosed GBM. Although the predictive and prognostic utilization of EGFR remains to be adequately defined, it has been reported that patients with GBM treated with temozolomide that had EGFR amplification, maintenance of PTEN, and wild-type p53 and p16 were strong prognostic indicators of overall survival [11].

### 2.6. Epidermal Growth Factor, Latrophilin, and 7 Transmembrane Domain-Containing Protein 1 on Chromosome 1 (*ELTD1*)

Immunochemistry was utilized to detect levels of ELTD1 in 50 human high-grade gliomas and rat F98 glioma tumors [12]. ELTD1 was found to be significantly higher (*p* = 0.03) in high-grade gliomas compared with low-grade gliomas (21 patients). In addition, *ELTD1* gene expression indicated an association with grade, survival across grade, and an increase in the mesenchymal subtype. High *in vivo* levels of ELTD1 were also found in rat F98 glioma tumors compared with normal brain tissue (*p* < 0.001) and thus may serve as an additional biomarker in preclinical and clinical diagnosis of gliomas.

### 2.7. Vascular Endothelial Growth Factor (VEGF)

Neovascularization is a neuropathological hallmark in high grade gliomas and angiogenic factors may play an important role in malignant tumor progression. VEGF is considered to be the driving factor of angiogenesis and has been identified in 64.1% GBMs, and a strong correlation between VEGF expression and survival has been reported indicating that VEGF is a potential prognostic factor in patients with gliomas [13].

### 2.8. p53

*TP53* is the gene that encodes the tumor suppressor protein, p53. A population-based study of 715 patients with a diagnosis of GBM has reported that the type and distribution of *TP53* mutations differs between GBM subtypes. In secondary GBMs, 57% of mutations were located in the two hotspot codons, 248 and 273 whereas in primary GBMs, mutations were more equally distributed through exons with only 17% occurring in codons 248 and 273, possibly reflecting increased genomic instability during tumor progression [28]. Currently, p53 is not known to be predictive or prognostic [14] but has a role in diagnosis as it can help to distinguish tumor grade.

### 2.9. *PTEN*

*PTEN* is a tumor suppressor gene that negatively regulates the phosphatidylinositol 3'-kinase (PI3K)/a serine/threonine kinase (Akt) pathway, and plays an important role in the regulation of cell proliferation, apoptosis, and tumor invasion [33]. In GBM, *PTEN* is deleted due to LOH of chromosome 10q in 50–70% of primary cases and 54%–63% of secondary GBM. It is also mutated in 14%–47% of primary GBM [33]. Currently, PTEN is thought to be a prognostic molecular marker as patients with loss of PTEN have decreased survival and may also be a possible predictive biomarker of glioma response to specific therapies [13].

### 2.10. p16INK4a

The p16INK4a gene binds to cyclin-dependent kinase 4 and inhibits the cyclin-dependent kinase 4-cyclin D1 complex. This complex phosphorylates the retinoblastoma protein (Rb), a tumor suppressor that is the central protein responsible for antiproliferative signaling. Deletions or mutations of the *Rb* gene occur in 40% of secondary GBM cases [27]. P16 loss has also been reported in 20%–57% of GBM cases [34]. Findings regarding the predictive value of p16INK4a homozygous deletion have been inconsistent [13] but a significant association between EGFR amplification and p16INK4a deletion has been reported [28].

### 2.11. Cytochrome c Oxidase

Cytochrome c Oxidase (CcO) is the terminal enzyme of the mitochondrial respiratory chain (electron transport chain) that catalyzes the transfer of electrons from cytochrome c to oxygen [15]. A recent retrospective study of 84 GBM patient tumors has reported that the median survival for patients with low tumor CcO was 14.3 months, compared with 6.3 months for patients with high tumor CcO activity. High CcO activity was found to be an independent predictor of poor outcome and may in the future become a useful molecular marker for the categorization of GBM and in development of targeted therapy [35].

### 2.12. Phospholipid Metabolites

It has been reported that metabolic changes during antiangiogenic therapy of recurrent GBM may provide new biomarkers for treatment efficacy [16]. *In vivo* magnetic resonance spectroscopic imaging of human recurrent GBMs before and during bevacizumab (Avastin; Genentech, Inc., South San Francisco, CA, USA) treatment was performed prospectively on 32 patients with recurrent GBM. Phosphocholine (PCho), phosphoethanolamine (PEth), glycerophosphocholine (GPC), and glyceroethanolamine (GPE) metabolite concentrations from tumor tissue and their ratios utilizing magnetic resonance spectroscopic imaging were compared to contralateral normal-appearing tissue (control). The short overall survival group (<median overall survival) showed higher PCho/GPC (*p* = 0.004) in recurrent GBMs compared to control tissue before bevacizumab, while PEth/GPE was elevated in recurrent GBMs in both short and long overall survival (>median overall survival) groups. During bevacizumab treatment, PCho/GPC and PEth/GPE in the tumor initially decreased (*p* = 0.04) but only PCho/GPC increased again on tumor progression (*p* = 0.02) indicating that an elevated PCho/GPC ratio in the short-overall survival group may be a negative predictive marker for bevacizumab efficacy; gliomas with this signature may represent an aggressive phenotype which grow despite bevacizumab therapy. Caution is advised in interpreting these data due to small numbers of patients involved.

### 2.13. Telomerase Messenger Expression (hTERT Messenger Ribonucleic Acid [mRNA])

Human telomerase is a structurally complex ribonucleoprotein that is responsible for the maintenance of telomeric DNA at the ends of chromosomes and is proposed as having an important role in cell immortalization and oncogenesis. A study which examined the telomerase activity by the telomeric repeat amplification protocol assay in 42 gliomas, which included 32 GBM samples, found that advanced age as well as high telomerase activity and hTERT mRNA levels were significant predictors of worse prognosis regarding both overall survival and disease-free survival [18]. Another study confirmed that hTERT was expressed in glioma cell lines and tissues tested but was absent in control cells and tissues and could thus potentially be useful as a biological diagnostic tool [17].

## 3. MicroRNAs (miRNAs)

MicroRNAs are small non-coding RNA molecules, approximately 21–25 nucleotides in length. More than 1,500 precursors and 1,921 mature *Homo sapien* miRNAs have been identified and registered in miRBase [36]. Deregulation of miRNAs has been implicated in GBM with reports of association of miRNA dysregulation with acquired temozolomide resistance and that miRNAs may play a role in cancer stem cell properties contributing to treatment resistance [37]. Many reports on the topic of MicroRNAs and their relationship with the clinicopathological features of GBM have recently been published. For example, one study examined expression profiles of miRNAs and genes and the corresponding clinical information of 480 GBM samples from The Cancer Genome Atlas dataset. Survival analysis showed that high levels of miRNA-326/miRNA-130a and low levels of miRNA-323/miRNA-329/miRNA-155/miRNA-210 were significantly associated with long overall survival of GBM patients, and also showed that high miRNA-326/miRNA-130a and low miRNA-155/miRNA-210 were related to extended progression free survival. Also, miRNA-323 and miRNA-329 were found to be increased in patients with no recurrence or a long time to progression [19]. Expression of miRNA-328 has been found to be decreased in 20 anaplastic and 60 GBM tumor samples and low miRNA-328 expression conferred poor survival in primary GBM patients [38]. Another study of 38 patients with primary GBM reported that miRNA-195 and miRNA-196b positively correlated with overall survival and that the combination of miRNA-181c and miRNA-21 predicted time to progression within six months of diagnosis with 92% sensitivity and 81% specificity and thus may identify patients at high risk of early progression after surgery [39]. Plasma levels of miRNAs, and specifically miRNA-21, miRNA-128 and miRNA-342-3p were also found to be altered in 50 patients with GBM compared to normal controls and miRNA-128 and miRNA-342-3p were found to be positively correlated with histopathological grades of glioma and suggest that plasma specific miRNAs could have potential use as biomarkers of glioma [40].

In addition, a significant anti-correlation between miRNA-18 and transforming growth factor β (TGFβ) signaling (signaling that has been found to be highly active in high grade gliomas and associated with poor clinical outcome), in primary GBM samples from the Cancer Genome Atlas has been reported. High levels of miRNA-18 combined with low levels of the TGFβ metagene correlated with prolonged patient survival and thus it was postulated that low expression of miRNA-18a may significantly contribute to GBM pathogenesis [41].

Therefore, miRNAs may be putative biological markers for diagnosis and possibly promising targets for GBM treatment in the future through establishment of miRNA-targeted therapies (based on miRNA dysregulation of cancer stem cells) in temozolomide resistant GBM. *In vitro* studies have revealed that chronic temozolomide exposure of the human GBM cell line D54MG resulted in acquired temozolomide resistance and elevated miRNA-21 expression. Concomitant treatment with a miRNA-21 inhibitor and temozolomide resulted in a significantly higher apoptotic rate than temozolomide alone. It was postulated that miRNA-21 may have potential for use as a biomarker of acquired temozolomide resistance and be a potential adjunct in the treatment of temozolomide resistant GBM [42]. All of these reports are investigational and further study in large populations of GBM tumor samples are required before application to the general clinical setting.

## 4. Cancer Stem Cells in GBM

There is growing evidence that cancer stem cells play an important role in tumor initiation, maintenance and recurrence [43]. These stem cell populations have been reported to be radio and chemo resistant [44]. However, the susceptibility of GBM cancer stem cells to standard treatment is controversial as existing literature is conflicting. The interaction of cancer stem cells and chemotherapy, for example, is complex as detoxifying proteins such as MGMT may confer intrinsic resistance to cancer stem cells. Extrinsic factors such as temozolomide concentrations in the brain parenchyma, temozolomide dosing schedules, hypoxic microenvironments, niche factors, and re-acquisition of stem cell properties by non-stem cells may also contribute [45]. Tumor suppressor p53 is frequently mutated in GBMs and appears to also have some contribution to resistance to temozolomide and other therapeutic drugs [46]. Tumor cell-originated neovascularization including tumor derived endothelial cell-induced angiogenesis and vasculogenic mimicry has also been suggested to be involved in the resistance to anti-VEGF therapy [47]. These factors have to be considered in the targeting of chemoresistance.

Identification of novel markers for GBM stem cells may lead to better targeting of these cells and thus may impact on the treatment of GBM. The CD133 epitope has been identified as a tumor marker for the purification of a subpopulation of GBM cells demonstrating cancer stem cell phenotypes, and in brain tumor xenografts simulates the heterogeneity of GBMs *in situ* [48]. However, the validity of CD133 as a cancer stem cell marker and hence its clinical ramifications remain controversial. One study reported that the CD133 gene signature identified an aggressive subtype of GBM seen in younger patients with shorter survival [21]. This same study also reported that the CD133 gene signature distinguished higher grade breast and bladder cancers from their lower grade counterparts. Additionally, Metellus *et al*. reported that high CD133 mRNA expression in a cohort of 48 consecutive primary GBM patients treated by chemoradiation with temozolomide, was a significant (*p* = 0.007) prognostic factor for adverse progression free and overall survival independent of resection (*p* = 0.012) and MGMT methylation status (*p* = 0.002) [20]. Another study reported that CD133 expression was not a predictive marker in GBM patients and analyses of possible correlation between CD133 expression and MGMT protein expression or MGMT promoter methylation were negative [44], therefore further studies are needed to determine the clinical utility of CD133.

Another study explored the potential role of CD90 as a marker for cancer stem cells in gliomas and immunofluorescence staining for CD90 and CD133 in 15 GBM tissues revealed that CD133(+) cancer stem cells are a subpopulation of CD90(+) cells in GBM *in vivo*. It was concluded that CD90 was not only a potential prognostic marker for high grade gliomas but also a marker for cancer stem cells within gliomas and it resides within endothelial niches and may play a role in generation of tumor vasculature via. differentiation into endothelial cells [49].

## 5. Imaging Modalities and Their Potential Biomarker Status

A recent retrospective study measured relative cerebral blood volume (rCBV) (maximum rCBV [rCBV(max)] and mean rCBV [rCBV(mean)]) and correlated it with patient survival and also determined its association with molecular subclasses of GBM [50]. Fifty patients had dynamic susceptibility contrast-enhanced T2-weighted magnetic resonance perfusion studies and had gene expression data available from the Cancer Genome Atlas. Patients were classified according to the four subclasses of GBM described by Verhaak *et al*. in 2010 [4] and the three subclasses of GBM described by Phillips *et al*. in 2006 [5]. Increased rCBV measures were found to be associated with poor overall survival in GBM with rCBV (max) (*p* = 0.0131) being the strongest predictor of overall survival regardless of potential confounders or molecular classification. Including the molecular GBM classification described by Verhaak *et al.* in the survival model clarified the association of rCBV (mean) with overall patient survival (hazard ratio: 1.46, *p* = 0.0212) compared with rCBV (mean) alone (hazard ratio: 1.25, *p* = 0.1918) suggesting that potentially molecular markers could be utilized in combination with hemodynamic imaging biomarkers in the future in an effort to predict patient overall survival.

**Figure 1:**
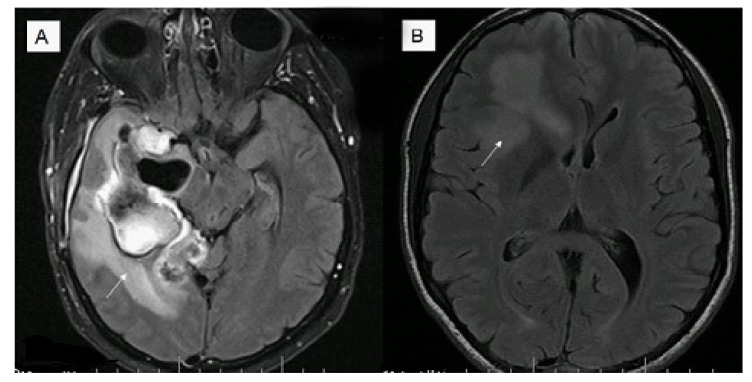
Magnetic resonance fluid attenuation inversion recovery (FLAIR) T2 imaging (**A**) of a 39 year old male, O(6)-methlyguanine-DNA-methyltransferase (MGMT) methylated glioblastoma (GBM) with increased edema (survival eight months post diagnosis); (**B**) 32 year old male, MGMT methylated GBM with less edema (alive seven years post diagnosis).

An additional study has investigated the relationship between tumor enhancement, edema, *IDH1* mutational status, *MGMT* promoter methylation, and survival in GBM [51]. Magnetic resonance imaging from 202 patients with GBM were retrospectively assessed for nonenhancing tumor and edema. All *IDH1* mutant tumors were non contrast enhancing and most were located in the frontal lobe (11/14, 79%) with larger tumor size. Edema stratified survival in *MGMT* promoter methylated but not in unmethylated tumors with median survival for methylated tumors with little/no edema being 2,476 days (95% confidence interval, 795) compared with 586 days (95% confidence interval, 507–654) for unmethylated tumors or tumors with edema (for example see Figure 1).

Conventional ^18^F-fluorodeoxyglucose (FDG)-positron emission tomography (PET) is of limited usefulness for imaging GBM. However, fluorothymidine (FLT) has shown promise as thymidine is taken up preferentially by proliferating cells. The effectiveness of ^18^F-FLT-PET was studied in a GBM mouse model, after radiation therapy, and its correlation with biomarkers of proliferation (Ki-67), DNA damage and repair (γH2AX), hypoxia (hypoxia inducible factor [HIF]-1α), and angiogenesis (VEGF) was investigated. It was shown that ^18^F-FLT-PET imaging reflected the inhibition of tumor by radiotherapy and correlated with changes in the biomarker expression [52] and may be a promising imaging modality in the future and potentially also be useful in the assessment of radiotherapy effects and biologically relevant biomarkers.

Predicting tumor response to anti-angiogenic therapy in individual patients is difficult. The predictive value of apparent diffusion coefficient (ADC) histogram analysis (generated from magnetic resonance imaging from areas of enhancing tumor on T1 weighted post-contrast images fitted to a two normal distribution curve) in stratifying progression-free survival and overall survival in bevacizumab-treated patients has been investigated in bevacizumab-treated patients with newly diagnosed GBM treated “up front” before tumor recurrence and in recurrent GBM patients. For up-front bevacizumab-treated patients (n = 59 *vs.* 62 control patients), lower ADC (L) was associated with significantly longer progression-free survival (median 459 days for ADC (L) <1,200 *vs*. 315 days for ADC (L) >/=1,200 10^−6^ mm^2^/s; *p* = 0.008, logrank test) and trended with longer survival (581 *vs*. 429 days, *p* = 0.055). Tumors with MGMT promoter methylation had lower ADC (L) values than unmethylated tumors [53]. The same group then compared gene expression between high and low ADC tumors to identify gene expression modules that may impact patient prognosis and in 38 “up front” bevacizumab-treated patients, it was reported that high ADC GBMs showed greater levels of extracellular matrix protein gene expression compared with low ADC GBMs, which could possibly contribute to a proinvasive phenotype [54].

In contrast, the predictive value of ADC histogram analysis was again reported in 97 bevacizumab-treated patients with recurrent GBM, but in this case, low ADC (L) was associated with poor outcome in a post-hoc analysis from the multi-center randomized, phase II BRAIN study evaluating bevacizumab with or without irinotecan in recurrent temozolomide treated GBM patients [55]. Another smaller study of 15 patients with recurrent GBM treated with bevacizumab and irinotecan reported prognostic significance of changes in parameters derived from diffusion tensor imaging (DTI). DTI detected a change in ADC within fluid attenuation inversion recovery (FLAIR) signal abnormality (FSA) in nine out of 15 patients (five in whom ADC was increased, four in whom it was decreased). Patients with a change in ADC within FSA had significantly shorter overall survival (*p* = 0.032) and progression-free survival (*p* = 0.046) than those with no change [56].

Tumor ADC value may be a useful marker in prediction of bevacizumab response and survival but larger studies are needed before this modality is universally accepted. A greater understanding is also required of the differences noted between changes seen following “up front” therapy (which is not standard of care in GBM), and in the recurrent setting.

## 6. Conclusions

Classical molecular markers with clinical implications such as MGMT promoter methylation, 1p 19q codeletion, and *IDH1* mutations are known favorable prognostic markers in gliomas, with *MGMT* promoter methylation being the only potential predictive marker for alkylating chemotherapy in GBM. While numerous other genetic alterations have been described in GBM as documented above, molecular and imaging biomarkers have proved to be of somewhat marginal utility in predicting outcome or guiding decisions about disease management. Currently, there are no significant differences in the treatment of GBM based on any molecular or imaging prognostic biomarkers. Additionally, reproducibility between studies of markers identified is unclear as is their validation for widespread clinical use.

As one example, genomic analysis has been used successfully for breast cancer stratification into molecular subgroups, with implications for clinical outcomes and detection of prognostic/treatment predictive signatures. Information derived from available assays allows clinicians to estimate risk for distant recurrence, and predict which patients are likely to benefit from adjuvant therapy [57]. However, to date, the incorporation of these molecular profiling technologies into clinical decision making is a slow process due to lack of accessibility and cost with some assays still requiring validation, but does illustrate that these tools may lead to better diagnostic, prognostic and therapeutic management in a clinically heterogenous and complex disease, and provides a standard which may eventually be applied to many other primary cancer diagnoses.

With increasing knowledge and thus molecular classification of GBM into a number of subgroups and their application to clinical practice, a greater understanding of the reasons for differing outcomes and the potential for individualized patient directed therapy will continue to evolve. Genotypic analysis is likely to become part of standard clinical care and it is important that all future trials incorporate genetic as well as histopathologic signature analysis to enable a greater appreciation of the pathogenesis of GBM, and to allow for more informed targeted therapy development.

## References

[1] Stupp R., Hegi M.E., Mason W.P., van den Bent M.J., Taphoorn M.J., Janzer R.C., Ludwin S.K., Allgeier A., Fisher B., Belanger K. (2009). Effects of radiotherapy with concomitant and adjuvant temozolomide versus radiotherapy alone on survival in glioblastoma in a randomized phase III study: 5-Year analysis of the EORTC-NCIC trial. Lancet Oncol..

[2] Polley M.Y., Lamborn K.R., Chang S.M., Butowski N., Clarke J.L., Prados M. (2011). Conditional probability of survival in patients with newly diagnosed glioblastoma. J. Clin. Oncol..

[3] Stupp R., Mason W.P., van den Bent M.J., Weller M., Fisher B., Taphoorn M.J., Belanger K., Brandes A.A., Marosi C., Bogdahn U. (2005). Radiotherapy plus concomitant and adjuvant temozolomide for glioblastoma. N. Engl. J. Med..

[4] Verhaak R.G., Hoadley K.A., Purdom E., Wang V., Qi Y., Wilkerson M.D., Miller C.R., Ding L., Golub T., Mesirov J.P. (2010). Integrated genomic analysis identifies clinically relevant subtypes of glioblastoma characterized by abnormalities in PDGFRA, IDH1, EGFR, and NF1. Cancer Cell.

[5] Phillips H.S., Kharbanda S., Chen R., Forrest W.F., Soriano R.H., Wu T.D., Misra A., Nigro J.M., Colman H., Soroceanu L. (2006). Molecular subclasses of high-grade glioma predict prognosis, delineate a pattern of disease progression, and resemble stages in neurogenesis. Cancer Cell.

[6] Jiao Y., Killela P.J., Reitman Z.J., Rasheed A.B., Heaphy C.M., de Wilde R.F., Rodriguez F.J., Rosemberg S., Oba-Shinjo S.M., Marie S.K.N. (2012). Frequent ATRX, CIC, FUBP1 and IDH1 mutations refine the classification of malignant gliomas. Oncotarget.

[7] Di Stefano A.L., Enciso-Mora V., Marie Y., Desestret V., Labussiere M., Boisselier B., Mokhtari K., Idbaih A., Hoang-Xuan K., Delattre J.Y. (2013). Association between glioma susceptibility loci and tumour pathology defines specific molecular etiologies. Neurooncology.

[8] Hegi M.E., Janzer R.C., Lambiv W.L., Gorlia T., Kouwenhoven M.C.M., Hartmann C., von Deimling A., Martinet D., Schmutz N.B., Diserens A.C. (2012). Presence of an oligodendroglioma-like component in newly diagnosed glioblastoma identifies a pathogenetically heterogenous subgroup and lacks prognostic value: Central pathology review of the EORTC_26981/NCIC_CE.3 trial. Acta Neuropathol..

[9] Schmidt M.C., Antweiler S., Urban N., Mueller W., Kuklik A., Meyer-Puttlitz B., Wiestler O.D., Louis D.N., Fimmers R., von Deimling A. (2002). Impact of genotype and morphology on the prognosis of glioblastoma. J. Neuropathol. Exp. Neurol..

[10] Jansen M., Yip S., Louis D.N. (2010). Molecular pathology in adult neuro-oncology: An update on diagnostic, prognostic and predictive markers. Lancet Neuro..

[11] Ang C., Guiot M.C., Ramanakumar A.V., Roberge D., Kavan P. (2010). Clinical significance of molecular biomarkers in glioblastoma. Can. J. Neurol. Sci..

[12] Towner R.A., Jensen R.L., Colman H., Vaillant B., Smith N., Casteel R., Saunders D., Gillespie D.L., Silasi-Mansat R., Lupu F. (2013). ELTD1, a potential new biomarker for gliomas. Neurosurgery.

[13] Bell E.H., Hadziahmetovic M., Chakravarti A., Abujamra A.L. (2011). Evolvement of molecular biomarkers in targeted therapy of malignant gliomas. Brain Tumors—Current and Emerging Therapeutic Strategies.

[14] Tabatabai G., Stupp R., van den Bent M.J., Hegi M.E., Tonn J.C., Wick W., Weller M. (2010). Molecular diagnostics of gliomas: The clinical perspective. Acta Neuropathol..

[15] Kadenbach B., Huttemann M., Arnold S., Lee I., Bender E. (2000). Mitochondrial energy metabolism is regulated via nuclear-coded subunits of cytochrome c oxidase. Free Radic. Biol. Med..

[16] Hattingen E., Bahr O., Rieger J., Blasel S., Steinbach J., Pilatus U. (2013). Phospholipid metabolites in recurrent glioblastoma: *In vivo* markers detect different tumor phenotypes before and under antiangiogenic therapy. PLoS One.

[17] Shervington A., Patel R., Lu C., Cruickshanks N., Lea R., Roberts G., Dawson T., Shervington L. (2007). Telomerase subunits expression variation between biopsy samples and cell lines derived from malignant glioma. Brain Res..

[18] Boldrini L., Pistolesi S., Gisfredi S., Ursino S., Ali G., Pieracci N., Basolo F., Parenti G., Fontanini G. (2006). Telomerase activity and hTERT mRNA expression in glial tumors. Int. J. Oncol..

[19] Qiu S., Lin S., Hu D., Feng Y., Tan Y., Peng Y. (2013). Interactions of miR-323/miR-326/miR-329 and miR-130a/miR-155/miR-210 as prognostic indicators for clinical outcome of glioblastoma patients. J. Transl. Med..

[20] Metellus P., Nanni-Metellus I., Delfino C., Colin C., Tchogandjian A., Coulibaly B., Fina F., Loundou A., Barrie M., Chinot O. (2011). Prognostic impact of CD133 mRNA expression in 48 glioblastoma patients treated with concomitant radiochemotherapy: A prospective patient cohort at a single institution. Ann. Surg. Oncol..

[21] Yan X., Ma L., Yi D., Yoon J.G., Diercks A., Flotz G., Price N.D., Hood L.E., Tian Q. (2011). A CD133-related gene expression signature identifies an aggressive glioblatoma subtype with excessive mutations. Proc. Natl. Acad. Sci. USA.

[22] Hegi M.E., Diserens A.C., Gorlia T., Hamou M.F., de Tribolet N., Weller M., Kros J.M., Hainfellner J.A., Mason W., Mariani L. (2005). MGMT gene silencing and benefit from temozolomide in glioblastoma. N. Engl. J. Med..

[23] Mason S., McDonald K. (2012). MGMT testing for glioma in clinical laboratories: Discordance with methylation analyses prevents the implementation of routine immunohistochemistry. J. Cancer Res. Clin. Oncol..

[24] Noushmehr H., Weisenberger D.J., Diefes K., Phillips H.S., Pujara K., Berman B.P., Pan F., Pelloski C.E., Sulman E.P., Bhat K.P. (2010). Identification of a CpG island methylator phenotype that defines a distinct subgroup of glioma. Cancer Cell.

[25] Shinawi T., Hill V.K., Krex D., Schackert G., Gentle D., Morris M.R., Wei W., Cruickshank G., Maher E.R., Latif F. (2013). DNA methylation profiles of long- and short-term glioblastoma survivors. Epigenetics.

[26] Riemenschneider M.J., Jeuken J.W., Wesseling P., Reifenberger G. (2010). Molecular diagnostics of gliomas: State of the art. Acta Neuropathol..

[27] Gladson C.L., Prayson R.A., Liu W.M. (2010). The pathobiology of glioma tumors. Annu. Rev. Pathol..

[28] Ohgaki H., Dessen P., Jourde B., Horstmann S., Nishikawa T., di Patre P.L., Burkhard C., Schuler D., Probst-Hensch N.M., Maiorka P.C. (2004). Genetic pathways to glioblastoma: A population-based study. Cancer Res..

[29] Fults D., Pedone C.A., Thompson G.E., Uchiyama C.M., Gumpper K.L., IIiev D., Vinson V.L., Tavtigian S.V., Perry W.L. (1998). Microsatellite deletion mapping on chromosome 10q and mutation analysis of MMAC1, FAS, and MXI1 in human glioblastoma multiforme. Int. J. Oncol..

[30] Wooten E.C., Fults D., Duggirala R., Williams K., Kyritsis A.P., Bondy M.L., Levin V.A., O’Connell P. (1999). A study of loss of heterozygosity at 70 loci in anaplastic astrocytoma and glioblastoma multiforme with implications for tumor evolution. Neurooncology.

[31] Kakkar A., Suri V., Jha P., Srivastava A., Sharma V., Pathak P., Sharma M.C., Sharma M.S., Kale S.S., Chosdol K. (2011). Loss of heterozygosity on chromosome 10q in glioblastomas, and its association with other genetic alterations and survival in Indian patients. Neurol. India.

[32] Fischer I., Aldape K. (2010). Molecular tools: Biology, prognosis, and therapeutic triage. Neuroimaging Clin. N. Am..

[33] Simpson L., Parsons R. (2001). PTEN: Life as a tumor suppressor. Exp. Cell Res..

[34] Kim B., Myung J.K., Seo J.H., Park C.K., Paek S.H., Kim D.G., Jung H.W., Park S.H. (2010). The clinicopathologic values of the molecules associated with the main pathogenesis of the glioblastoma. J. Neurol. Sci..

[35] Griguer C., Cantor A.B., Fathallah-Shaykh H.M., Gillespie G.Y., Gordon A.S., Markert J.M., Radovanovic I., Clement-Schatio V., Shannon C.N., Oliva C.R. (2013). Prognostic relevance of Cytochrome c Oxidase in primary Glioblastoma Multiforme. PLoS One.

[36] Kozomara A., Griffiths-Jones S. (2011). miRBase: Integrating microRNA annotation and deep-sequencing data. Nucleic Acids Res..

[37] Mizoguchi M., Guan Y., Yoshimoto K., Hata N., Amano T., Nakamizo A., Sasaki T. (2013). Clinical implications of microRNAs in human glioblastoma. Front. Oncol..

[38] Wu Z., Sun L., Wang H., Yao J., Jiang C., Xu W., Yang Z. (2012). MiR-328 expression is decreased in high-grade gliomas and is associated with worse survival in primary glioblastoma. PLoS One.

[39] Lakomy R., Sana J., Hankeova S., Fadrus P., Kren L., Lzicarova E., Svoboda M., Dolezelova H., Smrcka M., Vyzula R. (2011). MiR-195, miR-196b, miR-181c, miR-21 expression levels and o-6-methylguanine-DNA methyltransferase methylation status are associated with clinical outcome in glioblastoma patients. Cancer Sci..

[40] Wang Q., Pengcun L., Ailin L., Jiang W., Wang H., Wang J., Xie K. (2012). Plasma specific miRNAa as predictive biomarkers for diagnosis and prognosis of glioma. J. Exp. Clin. CancerRes..

[41] Fox J.L., Dews M., Minn A.J., Thomas-Tikhonenko A. (2013). Targeting of TGFβ signature and its essential component CTGF by miR-18 correlates with improved survival in glioblastoma. RNA.

[42] Wong S.T., Zhang X.Q., Zhuang J.T., Chan H.L., Li C.H., Leung G.K. (2012). MicroRNA-21 inhibition enhances *in vitro* chemosensitivity of temozolomide-resistant glioblastoma cells. Anticancer Res..

[43] Cho D.Y., Lin S.Z., Yang W.K., Hsu D.M., Lin H.L., Lee H.C., Lee W.Y., Chiu S.C. (2011). The role of cancer stem cells (CD133(+)) in malignant gliomas. Cell Transplant..

[44] Melguizo C., Prados J., Gonzalez B., Ortiz R., Concha A., Alvarez P.J., Madeddu R., Perazzoli G., Oliver J.A., Lopez R. (2012). MGMT promoter methylation status and MGMT and CD133 immunohistochemical expression as prognostic markers in glioblastoma patients treated with temozolomide plus radiotherapy. J. Transl. Med..

[45] Beier D., Schulz J.B., Beier C.P. (2011). Chemoresistance of glioblastoma cancer stem cells-much more complex than expected. Mol. Cancer.

[46] Chiang M.F., Chou P.Y., Wang W.J., Sze C.I., Chang N.S. (2013). Tumor suppressor WWOX and p53 alterations and drug resistance in glioblastomas. Front. Oncol..

[47] Soda Y., Myskiw C., Rommel A., Verma I.M. (2013). Mechanisms of neovascularization and resistance to anti-angiogenic therapies in glioblastoma multiforme. J. Mol. Med.(Berl).

[48] Jamal M., Rath B.H., Tsang P.S., Camphausen K., Tofilon P.J. (2012). The brain microenvironment preferentially enhances the radioresistance of CD133 (+) glioblastoma stem-like cells. Neoplasia.

[49] He J., Liu Y., Zhu T., Zhu J., Dimeco F., Vescovi A.L., Heth J.A., Muraszko K.M., Fan X., Lubman D.M.  (2012). CD90 is identified as a candidate marker for cancer stem cells in primary high-grade gliomas using tissue microarrays. Mol. Cell. Proteomics.

[50] Jain R., Poisson L., Narang J., Gutman D., Scarpace L., Hwang S.N., Holder C., Wintermark M., Colen R.R., Kirby J. (2013). Genomic mapping and survival prediction in glioblastoma: Molecular subclassification strengthened by hemodynamic imaging biomarkers. Radiology.

[51] Carrillo J.A., Lai A., Nghiemphu P.L., Kim H.J., Phillips H.S., Kharbanda S., Moftakhar P., Lalaezari S., Yong W., Ellingson B.M. (2012). Relationship between tumor enhancement, edema, IDH1 mutational status, MGMT promoter methylation, and survival in glioblastoma. AJNR Am. J. Neuroradiol..

[52] Chandrasekaran S., Hollander A., Xu X., Benci J.L., Davis J.J., Dorsey J.F., Kao G. (2013). 18F-fluorothymidine-PET imaging of glioblastoma multiforme: Effects of radiation therapy on radiotracer uptake and molecular biomarker patterns. Scientific World J..

[53] Pope W.B., Lai A., Mehta R., Kim H.J., Qiao J., Young J.R., Xue X., Goldin J., Brown M.S., Nghiemphu P.L. (2011). Apparent diffusion coefficient histogram analysis stratifies progression-free survival in newly diagnosed bevacizumab-treated glioblastoma. AJNR Am. J. Neuroradiol..

[54] Pope W.B., Mirsadraei L., Lai A., Eskin A., Qiao J., Kim H.J., Ellingson B., Nghiemphu P.L., Kharbanda S., Soriano R.H. (2012). Differential gene expression in glioblastoma defined by ADC histogram analysis: Relationship to extracellular matrix molecules and survival. AJNR Am. J. Neuroradiol..

[55] Pope W.B., Qiao X.J., Kim H.J., Lai A., Nghiemphu P., Xue X., Elingson B.M., Schiff D., Aregawi D., Cha S. (2012). Apparent diffusion coefficient histogram analysis stratifies progression-free and overall survival in patients with recurrent GBM treated with bevacizumab: A multi-center study. J. Neurooncol..

[56] Paldino M.J., Desjardins A., Friedman H.S., Vredenburgh J.J., Barboriak D.P. (2012). A change in the apparent diffusion coefficient after treatment with bevacizumab is associated with decreased survival in patients with recurrent glioblastoma multiforme. Br. J. Radiol..

[57] Gokmen-Polar Y., Badve S. (2012). Molecular profiling assays in breast cancer: Are we ready for prime time?. Oncology.

